# The Second Edition of Motivations Associated with Food Choices and Eating Practices

**DOI:** 10.3390/foods12162986

**Published:** 2023-08-08

**Authors:** Raquel P. F. Guiné

**Affiliations:** CERNAS-IPV Research Centre, Polytechnic University of Viseu, 3504-510 Viseu, Portugal; raquelguine@esav.ipv.pt

## 1. Introduction

Eating patterns are linked with both environmental and behavioural factors. Cultural issues, like poor access to information on the nutritional quality of foods and the influence of peers and friends towards eating healthy foods or towards eating fast-food and caloric-dense food, are certainly drivers for the food choices that determine eating habits [1].

Inappropriate eating leads to several health impairments and chronic diseases like obesity, diabetes, and heart-related diseases, among others. The mental state is undoubtedly a factor that strongly influences emotional eating, most of the time leading to overeating or eating dense foods of low nutritional quality, very high in fat and sugar and low in micronutrients (such as vitamins and minerals) or bioactive compounds. Cardoso et al. [2] studied perceptions about healthy eating and emotional factors that conditioned eating behaviour among Portuguese and Argentinian participants. They concluded that education was the most important discriminant sociodemographic variable related to perceptions about healthy eating, followed by professional area and country. On the other hand, the most relevant discriminants for the emotional conditioning of eating behaviour were country, followed by living environment and sex. The work by Bartkiene et al. [3] focused on several factors affecting consumer food choices (social status, age, gender, education, knowledge about healthy eating, and attitude to food), as well as the relationships between food taste, food choice and depression. They used sensory traits and face-reading technology, and their results showed that face-reading technology could accurately detect differences in facial expressions induced by different tastes of food for groups with and without depressive disorders. Bearing in mind the burden of obesity and the effect of emotional issues related to inappropriate eating practices, some current therapeutic approaches recognise the necessity of including emotional management and independence when choosing what to eat as part of successful treatment strategies. In this context, meditation has been studied and is presently recommended as a component of therapeutic management [1]. The study by Oliveira et al. [1] showed that meditation positively influenced weight loss, diminishing the levels of uncontrolled eating and reducing the intensity of dysfunctional eating behaviours. Additionally, anxiety levels were reduced, promoting a higher quality of life.

Eating practices are highly variable through the life phases, starting from young to old (children, adolescents, young adults, adults, and older people), because their social environments and relationships change, and their emotional maturity and life experiences change while growing old. The societal and cultural aspects as well as environmental concerns are also important factors that influence people’s eating practices. Modern trends to consume more sustainable diets have helped citizens to shift their diets according to sustainable development principles. The work by Kumar et al. [4] highlights the connection between agriculture, food environments, and nutritional outcomes, giving insightful hints to guide policy actions towards achieving food security and nutrition in rural regions in low- and middle-income countries where food environments are quickly changing. The work by Guiné et al. [5] with participants from 16 countries (Argentina, Brazil, Croatia, Egypt, Greece, Hungary, Italy, Latvia, Lithuania, Netherlands, Poland, Portugal, Serbia, Slovenia, Romania, United States of America), showed that participants tend to shape their food choices according to two types of concerns: purely environmental concerns and sustainability allied to quality concerns.

The study by Bélanger et al. [6] reports that constructs and beliefs from the theory of planned behaviour influence the motivation to adopt a healthy diet. Carbonneau et al. [7], reporting the results from the PREDISE study which focused on the motivational orientations for the regulation of eating as defined by self-determination theory and their association with sociodemographic characteristics and overall diet quality, confirm that some people tend to have more self-determined motivation for eating, thus resulting in better diet quality. Furthermore, the authors concluded that this improved diet was independent of people’s sociodemographic characteristics as well as of other individual and social determinants of healthy eating.

Personalized nutrition has been gaining importance over the years, using an approach centred on the person’s characteristics and lifestyle. People have been increasingly recognizing the concept of dietary health, and therefore the on-the-spot monitoring of food safety and nutrition, tracing the source of food, and individualized guidance on nutritional and healthy eating habits are becoming more relevant. In relation to this matter, the use of smartphones and programs with specific functions is expected to change the interface between the consumer and food choices. Three types of smartphone applications in the food arena can be distinguished: rapid food detection, food traceability systems, and personalized diet guidance [8], and they can have a positive effect in shaping food choices towards healthier dietary practices.

## 2. Some Developments in the Motivations that Determine People’s Eating Habits

In a context of rising world population, and considering that food is fundamental for the survival of humans, there is a growing worry among consumers about accessing foods of nutritional quality but produced with a minimum impact on the environment. It has been discussed that the new demands for food and animal protein have a high impact on climate change and the environment. However, these harmful impacts can be reduced through the transition to healthier and more sustainable diets. In recent years, edible insects have been highly recommended as an alternative source of protein, being more sustainable when compared with other sources of animal protein. In this context, the review by Florença et al. [9] focused on the motivations for consumption of edible insects. In their work, they reported that the principal motivations for the consumption of edible insects are associated with a variety of factors: some sociodemographic characteristics like sex or age; some environmental concerns, specifically related with sustainability; the quality of foods, and especially the nutritional value and sensory attributes; and finally with personal traits and cultural influences: namely tradition/culture, food neophobia, disgust and familiarity/past experiences. Their work also revealed that while in insect-eating countries there is a higher focus on factors related to sensory attributes, availability, affordability and preferences; in Western countries, the emphasis is more on the nutritional value, sustainability, benefits, familiarity/past experience, tradition/culture, food neophobia and disgust. Additionally, they found that in Western countries, there is a trend for higher willingness to accept foods that contain insects as part of their formulation instead of consuming the whole insect.

In a scenario of intensification of global warming, consumers tend to shape their eating patterns whilst also focusing on improving the environment. Considering that a high percentage of greenhouse gases from agriculture come from animal husbandry and that reducing the meat production can greatly impact the reduction in greenhouse gases, the transition from non-vegetarian to vegetarian diets based on alternative foods such as plant-based meat alternatives constitutes a way for consumers to gradually reduce their dependence on meat protein. Ma and Chang [10] applied the value–attitude–behaviour model (VAB) to investigate the attitudes and behaviour of consumers towards novel and environmentally friendly foods, namely plant-based meat alternatives. In their study, they used the novelty of these meat alternatives as an intervening variable in the model for discussion. Their results showed that perceptions of ‘green value’ and ‘animal welfare value’ had a significantly positive influence on consumer’s attitudes. On the other hand, attitudes and knowledge about the product also were found to have a significant positive effect on behaviour. Nevertheless, the novelty of the plant-based meat alternatives was not found to be an influential variable on the relationship between product knowledge and behaviour.

Presently, consumers tend to choose more natural and environmentally friendly foods, also preferring products that enhance their health status when making purchasing decisions. Consumers attend to the origin and the brand as important drivers for decision making, and acceptance is facilitated for more familiar products. The development of organic agriculture has been promoted worldwide as a way to reduce the impact on ecosystems and also as a way to improve the nutritional health of consumers. Ayaviri-Nina et al. [11] investigated the consumer behaviour towards the purchase of organic farming foods in Ecuador. By applying linear regression, they were able to identify which demographic and cultural factors determine consumer buying behaviour towards organic products. In this way, it was found that motivation, emotions, and feelings are significantly related to the consumer’s attitude towards and purchase of organic products.

Emotions are highly conditioning of food consumption and they can reflect in the perceptions of eating. An increase in food intake during emotional and psychological conditions may negatively impact the human health. Ljubičić et al. [12] evaluated emotional eating behaviour in 12 European countries (Croatia, Greece, Hungary, Italy, Latvia, Lithuania, the Netherlands, Poland, Portugal, Romania, Serbia, and Slovenia). Using regression models, the authors confirmed the associations between food consumption, emotional conditions, and emotional eating behaviour. Specifically, they found associations between the emotional eating behaviour and stress, depressive mood, loneliness, boredom, and emotional consolation. Furthermore, the authors observed that emotional eating was related with the desire to improve physical status and psychological wellbeing, namely in terms of controlling body weight, maintaining a more alert state and eating as a way to feel good. They concluded that emotions can in fact lead to emotional eating behaviour.

Traditional food products constitute an important trace of the culture, identity, and heritage of populations. These products are prepared using specific ingredients and special preparation methods, which have been passed down from generation to generation, and that, for these reasons, constitute part of the cultural heritage. Pivarski et al. [13] conducted a study to investigate the factors that influence the consumption of traditional food products in tourism, and for that, they focused on the perspective of employees in food and beverage catering facilities. Their work resulted in a scale considered appropriate to measure key factors that influence the consumption of traditional products used in catering facilities that offer meals to tourists. The results further showed that economic aspects are of pivotal importance in the consumption of traditional products.

In recent years, there has been a strong trend for the consumers to adopt healthier lifestyles and to convert to healthier eating patterns. To this end, adequate information and labelling on food packaging is an essential tool to help consumers make more informed dietary choices as a way to improve the quality of their diets and prevent some food-related diseases. The front-of-pack labelling is envisaged as one of the important tools to help consumers make healthier food choices. Panczyk et al. [14] studied the front-of-pack labelling in foods on sale in the Polish market, specifically the nutri-score nutritional label. The results revealed that the most relevant characteristics of a front-of-pack labelling system include clarity, simplicity, consistency with healthy eating recommendations, and the ability to objectively compare products within the same group. From their work, they observed that more than half of the participants considered that the Nutri-Score gives an easy global assessment of a food’s nutritional value, thus allowing rapid purchasing decisions. However, this system was found to be poor in some aspects, such as being insufficient to help consumers compose a balanced diet, and not being able to be applied to all product groups. Finally, some other fragilities were also pointed out, such as the system’s inability to show the food’s degree of processing, as well as not providing the full nutritional value and its associated carbon footprint.

People suffering from some diseases have metabolic alterations that make it imperative to associate therapy with certain specific dietary guidelines. Carvalhal et al. [15] evaluated the relationship between determinants of food choice and some socioeconomic and demographic variables in people suffering from hepatitis in the Amazon region. They used the Eating Motivation Survey (TEAMS) composed of 15 different sub-scales. Their results showed positive weak correlations between sub-scale ‘preference’ and the variables, gender and age. The sub-scale ‘price’ was negatively correlated with the variables, age and income. The sub-scale ‘emotion control’ was negatively correlated with age. Sub-scales ‘convenience’ and ‘social norms’ were both negatively correlated with education. The sub-scale ‘weight control’ was positively correlated with income. These results may be helpful in developing more realistic and feasible eating strategies for this type of patient.

## 3. Concluding Remarks

The shaping of one’s eating practices is highly variable according to a high number of motivations, with factors arising from the individual’s surroundings as well as personal traits and behaviours. Among the environmental drivers of food choice stand factors accounting for cultural, political, environmental, and social influences together with those from peers or family. Regarding the behavioural factors, personal preferences, mood, health status, and their relation with food cravings definitely have to be taken into account as being very relevant for food choice decisions.

## References

[1] Oliveira J.P.T., do Carmo S.G., Aragão B.d.A., Cunha J., Botelho P.B. (2023). Meditation Practices and Their Relationship with Eating Behavior, Weight Changes, and Mental Health in Adults from Different Regions of Brazil: A Cross-Sectional Study. Nutrition.

[2] Cardoso A.P., Ferreira V., Leal M., Ferreira M., Campos S., Guiné R.P.F. (2020). Perceptions about Healthy Eating and Emotional Factors Conditioning Eating Behaviour: A Study Involving Portugal, Brazil and Argentina. Foods.

[3] Bartkiene E., Steibliene V., Adomaitiene V., Juodeikiene G., Cernauskas D., Lele V., Klupsaite D., Zadeike D., Jarutiene L., Guiné R.P.F. (2019). Factors Affecting Consumer Food Preferences: Food Taste and Depression-Based Evoked Emotional Expressions with the Use of Face Reading Technology. BioMed Res. Int..

[4] Kumar S., Das A., Kasala K., Ridoutt B., Patan E.K., Bogard J., Ravula P., Pramanik S., Lim-Camacho L., Swamikannu N. (2023). Assessing the Rural Food Environment for Advancing Sustainable Healthy Diets: Insights from India. J. Agric. Food Res..

[5] Guiné R., Ferreira M., Correia P., Leal M., Ferreira V., Rumbak I., El-Kenawy A., Papageorgiou M., Szucs V., Vittadini E. (2020). Food Choices as Influenced by Environmental Concerns: Study Involving Participants from 16 Countries. J. Secur. Sustain. Issues.

[6] Bélanger M., Dugas C., Perron J., St-Yves A., Rancourt-Bouchard M., Weisnagel S.J., Robitaille J. (2023). Intention to Adopt a Healthy Diet among Women with and without a History of Gestational Diabetes: Constructs and Beliefs from the Theory of Planned Behavior. Prev. Med. Rep..

[7] Carbonneau E., Pelletier L., Bégin C., Lamarche B., Bélanger M., Provencher V., Desroches S., Robitaille J., Vohl M.-C., Couillard C. (2021). Individuals with Self-Determined Motivation for Eating Have Better Overall Diet Quality: Results from the PREDISE Study. Appetite.

[8] Ma T., Wang H., Wei M., Lan T., Wang J., Bao S., Ge Q., Fang Y., Sun X. (2022). Application of Smart-Phone Use in Rapid Food Detection, Food Traceability Systems, and Personalized Diet Guidance, Making Our Diet More Health. Food Res. Int..

[9] Florença S.G., Guiné R.P.F., Gonçalves F.J.A., Barroca M.J., Ferreira M., Costa C.A., Correia P.M.R., Cardoso A.P., Campos S., Anjos O. (2022). The Motivations for Consumption of Edible Insects: A Systematic Review. Foods.

[10] Ma C.-C., Chang H.-P. (2022). The Effect of Novel and Environmentally Friendly Foods on Consumer Attitude and Behavior: A Value-Attitude-Behavioral Model. Foods.

[11] Ayaviri-Nina V.D., Jaramillo-Quinzo N.S., Quispe-Fernández G.M., Mahmud I., Alasqah I., Alharbi T.A.F., Alqarawi N., Carrascosa C., Saraiva A., Alfheeaid H.A. (2022). Consumer Behaviour and Attitude towards the Purchase of Organic Products in Riobamba, Ecuador. Foods.

[12] Ljubičić M., Matek Sarić M., Klarin I., Rumbak I., Colić Barić I., Ranilović J., Dželalija B., Sarić A., Nakić D., Djekic I. (2023). Emotions and Food Consumption: Emotional Eating Behavior in a European Population. Foods.

[13] Kalenjuk Pivarski B., Tekić D., Šmugović S., Banjac M., Novaković A., Mutavdžić B., Ivanović V., Tešanović D., Đerčan B., Ikonić P. (2023). Factors Affecting the Consumption of Traditional Food in Tourism—Perceptions of the Management Sector of Catering Facilities. Foods.

[14] Panczyk M., Dobrowolski H., Sińska B.I., Kucharska A., Jaworski M., Traczyk I. (2023). Food Front-of-Pack Labelling and the Nutri-Score Nutrition Label—Poland-Wide Cross-Sectional Expert Opinion Study. Foods.

[15] Carvalhal M.M.d.L., da Silva R.M.J., Pereira T.C., Monteiro C.R., Gomes D.L., Quaresma J.A.S. (2023). Relationship between Determinants of Food Choices and Socioeconomic and Demographic Factors of Individuals with Hepatitis B and C in the Amazon Region. Foods.

